# Erythrodermic flare‐up of psoriasis with COVID‐19 infection: A report of two cases and a comprehensive review of literature focusing on the mutual effect of psoriasis and COVID‐19 on each other along with the special challenges of the pandemic

**DOI:** 10.1002/ccr3.5722

**Published:** 2022-04-20

**Authors:** Elham Behrangi, Afsaneh Sadeghzadeh‐Bazargan, Nastaran Salimi, Zoha Shaka, Mohammad Hosein Feyz Kazemi, Azadeh Goodarzi

**Affiliations:** ^1^ Department of Dermatology Rasool Akram Medical Complex Clinical Research Development Center (RCRDC) School of Medicine Iran University of Medical Sciences (IUMS) Tehran Iran; ^2^ 440827 School of Medicine Iran University of Medical Sciences Tehran Iran; ^3^ Network of Immunity in Infection, Malignancy and Autoimmunity (NIIMA) Universal Scientific Education and Research Network (USERN) Tehran Iran; ^4^ 48464 School of Medicine Kermanshah University of Medical Sciences Kermanshah Iran

**Keywords:** aggravation, biologics, case reports, corona virus, COVID‐19, disease course, erythroderma, erythrodermic psoriasis, flare‐up, immunomodulator, immunosuppressant, management, psoriasis, review, SARS‐CoV‐2, treatment

## Abstract

The COVID‐19 pandemic has been extra challenging for patients with chronic diseases. Psoriasis is one of the chronic conditions that its treatment mostly relies on immunosuppressants. In this study, we report two cases with a long history of psoriasis that COVID‐19 infection caused them to undergo erythrodermic psoriasis.

## Graphical abstract

In psoriatic patients, the administration of glucocorticoids during the course of COVID‐19 infection might affect patient's psoriasis presentation, as in some cases early after administrating steroids, psoriatic lesions seem to be mitigated, and after finishing the course of treatment, some patients have reported to experience certain degrees of psoriasis flare‐up.

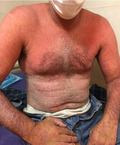

## 1 INTRODUCTION

Since the emergence of the novel COVID‐19 virus, chronic health conditions have been vastly impacted either directly through the infection and the immune responses followed by it or indirectly by imposing fear on some patients and causing them to restrict or delay their routine follow‐up cares, which might aggravate their condition.^1^, ^2^ Erythrodermic psoriasis is a severe and scarce form of psoriasis vulgaris that affects the entire body and provokes systemic inflammation with unknown certain etiology in which interleukin‐4, interleukin‐13, and TNF alpha have been suggested to have prominent roles.^3^ Here, we present two cases of psoriatic patients that developed symptoms of erythrodermic psoriasis after affliction with COVID‐19.

## 2 CASE PRESENTATION

### 2.1 Case 1

A 50‐year‐old male with a 20‐year history of psoriasis vulgaris was presented with erythrodermic psoriasis to the emergency department of our hospital. He was a retired employee, and his BMI was 28.68, had no other underlying health condition, and had been a cigarette smoker for 21 years (42 pack‐years) but had quit smoking 8 years ago and mentioned occasional consumption of alcohol. One month prior to the admission, he had been in close contact with a COVID‐19‐positive patient and developed fever and chills, fatigue, and myalgia shortly after. Real‐time PCR test was not carried out on, but in the chest CT scan, the typical signs of COVID‐19 infection were evident.

**FIGURE 1:**
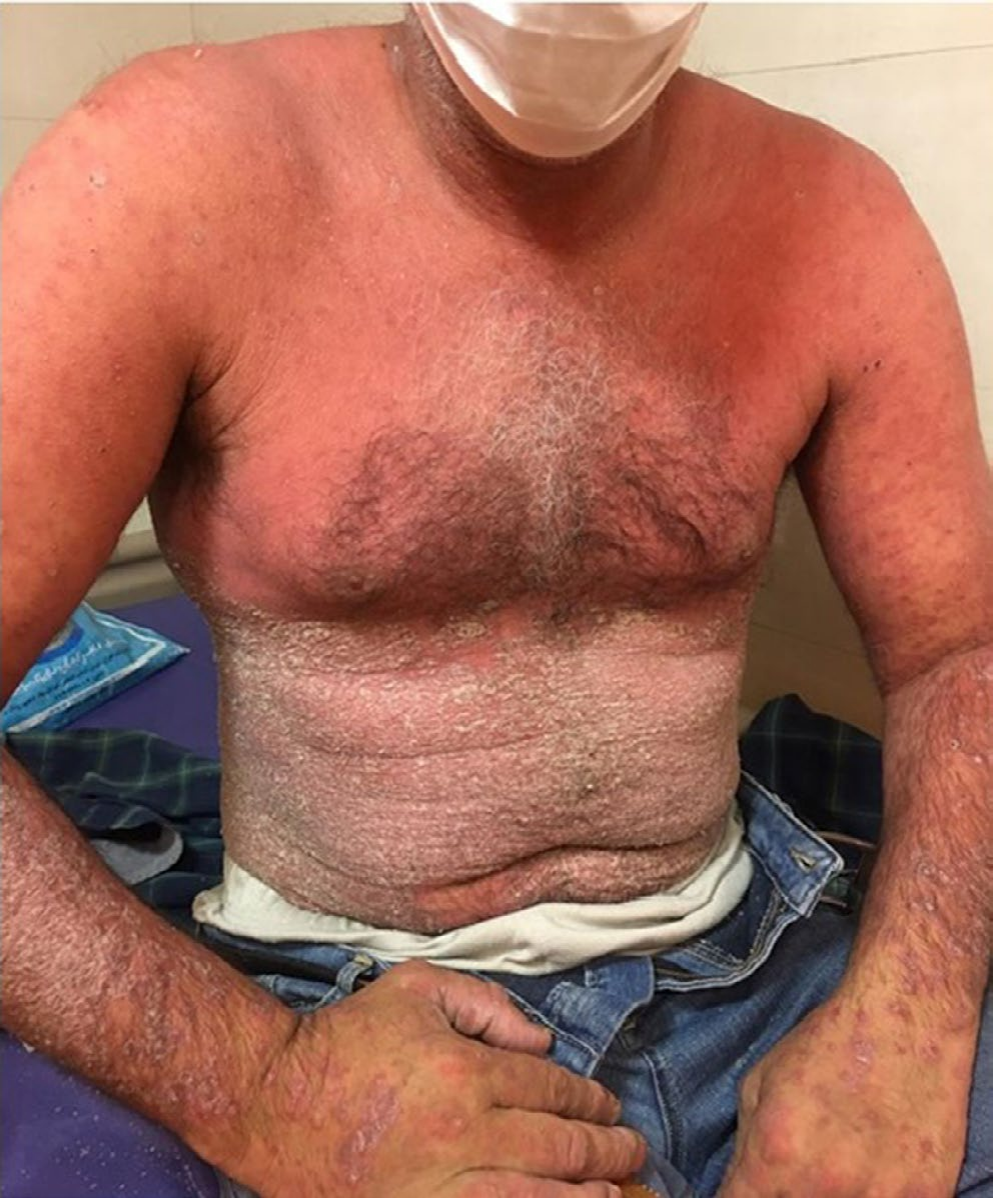
A 50‐year‐old male with generalized erythrodermic flare‐up of psoriasis 1 month after COVID‐19 infection, trunk lesions

**FIGURE 2:**
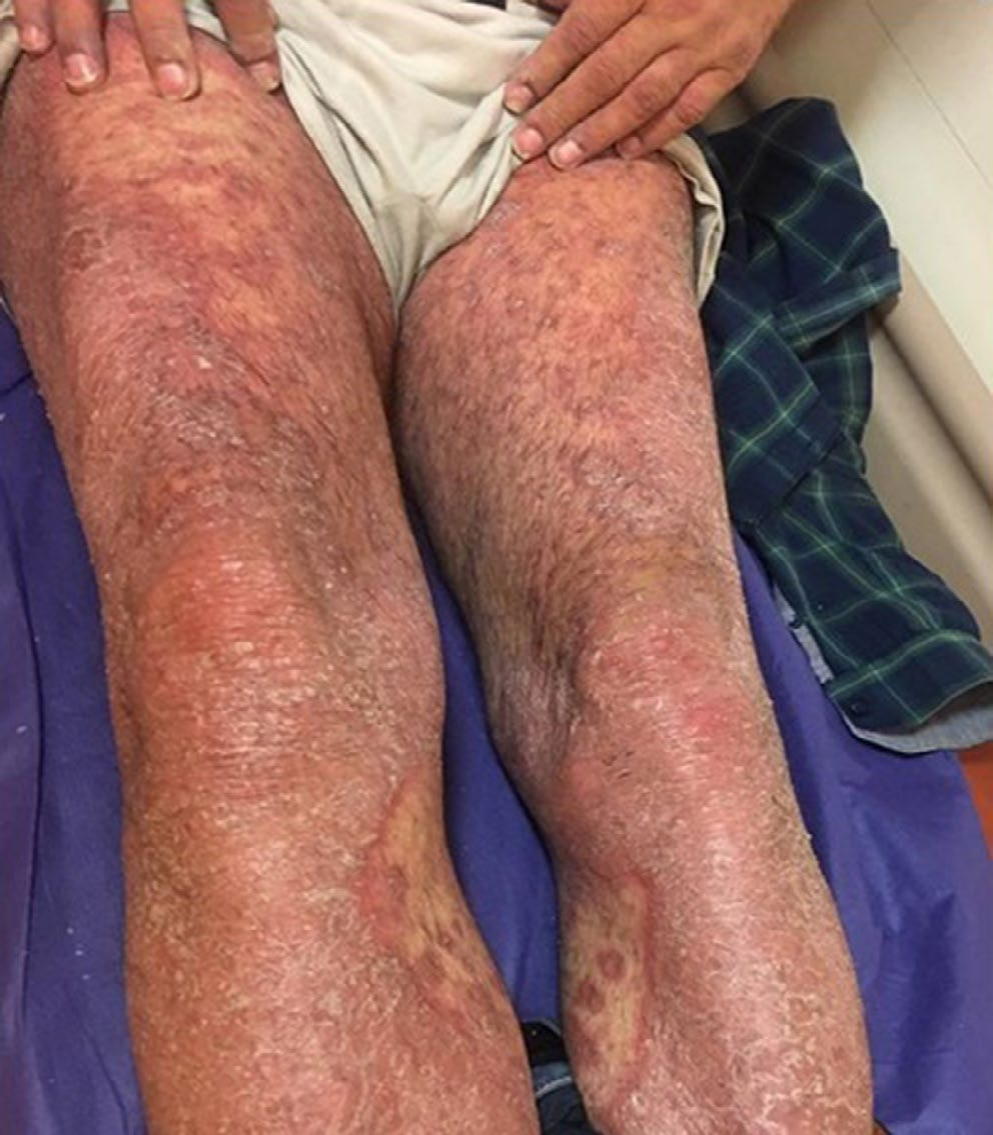
Same 50‐year‐old male patient with generalized erythrodermic flare‐up of psoriasis 1 month after COVID‐19 infection, lower extremities lesions

He had quarantined himself at home for 2 weeks and began taking Naproxen 250 mg tablet (three times a day), Hydroxychloroquine 500 mg tablet (twice a day), and N‐acetyl cysteine effervescent (daily) for 5 days and then without consulting a doctor added Amoxicillin 500 mg (three times a day) and Azithromycin 250 mg (daily) to his regimen. He had also delayed receiving Adalimumab due to his COVID‐19 infection. The patient stated that 2 days after taking the antibiotics, he developed general erythema and pruritus scaling that started from his palms and the soles of his feet with severe paresthesia. The physical examination revealed fever, weakness, and facial edematous accompanied by generalized skin redness and desquamation. (Figures 1 and 2) No signs of tachycardia, mucosal involvement, or swollen ankles were detected, and the rest of his examination was unremarkable. He had been a known case of psoriasis for about 20 years, he had been receiving Infliximab until 4 years ago, and his condition had been under control but after Infliximab was no longer covered by insurance, his medication was switched to Adalimumab, which relatively controlled his condition with occasional mild flare‐ups. The patient started receiving Methotrexate 10 years ago, but 6 months ago, it was discontinued due to hepatic cirrhosis. Due to his COVID‐19 infection, he had skipped one session of his Adalimumab therapy, which might have been a reason to this sudden flare‐up. Laboratory evaluation was significant for high lactic acid dehydrogenase (LDH = 549, normal range 140–280 U/L), and the rest of his laboratory results were within normal range Table 1.

Based on his clinical evaluation, the clinical diagnosis of severe erythrodermic psoriasis was made for him. After initial managements of his condition, he was treated with Cyclosporine 10 mg tablet (three times a day), Prednisone 50 mg tablet (daily), Neostigmine 25 mg capsule (twice a day), Acyclovir 80 mg tablet (three times a day), topical ointment of Mometasone (twice a day), and a mixed lotion of Eucerin and fluticasone (twice a day) on his lesions. He was discharged in a good condition and symptom free. Up to the time of this report, he has not experienced any psoriasis flare‐ups or hospitalization.

### 2.2 Case 2

**FIGURE 3:**
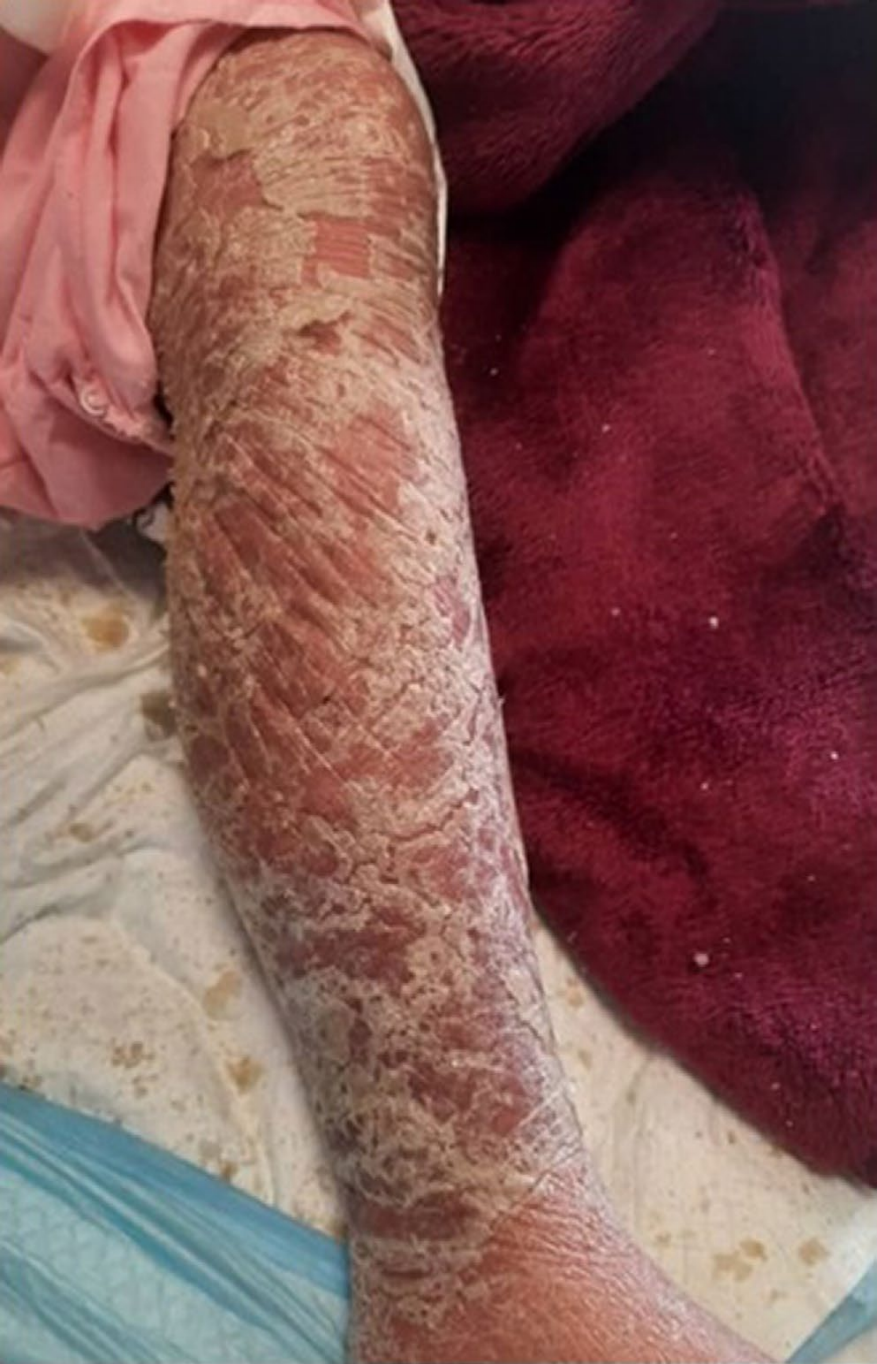
A 57‐year‐old female COVID‐19 patient with severe erythrodermic psoriasis

A 57‐year‐old female with a history of diabetes, hepatic cirrhosis, and psoriasis (for 15 years) was admitted to the internal medicine ward of our hospital with generalized edema and ascites along with general erythrodermic psoriasis legions on her trunk, face, upper, and lower limbs (Figure 3). She was isolated from other patients because despite her nontypical symptoms, she was suspected to be infected with COVID‐19.

We visited the patient for dermatology consultation. She was a housewife, and her BMI was 27.18. For diabetes, she had been receiving insulin, and for psoriasis, she had been receiving MTX for more than 10 years until being diagnosed with hepatic cirrhosis a few months ago. She had not been receiving any other substitutes for psoriasis ever since. She mentioned no history of smoking or alcohol consumption. The infectious disease department consulted us to refrain from administering Adalimumab or any other immunosuppressant. Therefore, we tried to manage her legions with topical agents (including Betamethasone and Vaseline ointment and Clobetasol lotion). A PCR test was obtained from her and confirmed her COVID‐19 infection, and she started receiving the routine COVID‐19 treatment according to national guidelines at the time. Unfortunately, her COVID‐19 condition was rapidly deteriorated and she was admitted to the intensive care unit and she expired.  Table 1 shows the summarized results for the cases presented in the literature focusing on mutual effect of psoriasis and COVID‐19 on each other.

**TABLE 1:** Comprehensive review of literatures focusing on mutual effect of psoriasis and COVID‐19 on each other

	Ref	Case characteristics	Drug history for psoriasis	Psoriasis condition	COVID−19 clinical features	Other comorbidities	COVID−19 progression	Psoriasis changes during the infection	COVID−19treatment	Psoriasis drug adjustment	Recommendations
COVID−19 in known psoriasis patients without specific changes in psoriatic symptoms (51)	^41^	A 55‐year‐old male	Conventional drugs + adalimumab switched to ixekizumab	Controlled psoriasis for 4 years + a recent flare‐up	Asymptomatic outpatient with positive PCR	Not mentioned	Stayed asymptomatic	No change in psoriatic features	Supportive therapy, home rest	Continued biological therapy as formerly prescribed	Biological therapy had positively affected the primary phase of infection
	^5^	A 40‐year‐old female	Systematic methotrexate and cyclosporine, then switched to Guselkumab for last 2 years	Controlled psoriasis since 2000	Severe cough, myalgia, fatigue, fever (39.4°C)	Ehlers–Danlos syndrome	Rapid worsening of symptoms, but sudden improvement after the Guselkumab injection	Not mentioned	Supportive therapy, home rest	Continued biological therapy as formerly prescribed	Biologic therapy tapered down the immune reaction
	^9^	An individual in their 30 s	Adalimumab for the last 6 months	Controlled psoriasis since childhood with widespread plaque	Symptomatic Outpatient with positive PCR, sore throat, fever (to 101.5 F), mild dry, cough, nonspecific gastrointestinal upset	Sleep apnea, chronic nerve pain	Week 1: recovering well Week 2: mild shortness of breath, respiratory discomfort, fatigue, and diaphoresis Week 3: at the end of 3rd week became symptom‐free	Not mentioned	Supportive therapy, home rest	Held biological therapy until being symptom free for 1 month	Patients on biologic therapies had successfully recovering of COVID−19 infection
	^9^	An individual in their 40 s	Ustekinumab for 3 years	Uncontrolled psoriasis for over 20 years with generalized plaque	Symptomatic outpatient with positive PCR, fever (100.8 F), fatigue, shortness of breath, mild nausea	Not mentioned	Gradually recovered and was symptom free in 9 days	Not mentioned	Supportive therapy, home rest	Held biological therapy until being symptom free for 1 month	Not mentioned
	^42^	A 62‐year‐old male	Guselkumab for past 2 years	Controlled psoriasis	Severe acute respiratory syndrome	HTN, DM, chronic renal failure and overweight (BMI: 29)	Hospitalization for 1 month (ICU admission for 2 weeks) then discharged symptom free	Not mentioned	Hospitalization and intensive care	Held biological therapy	Not mentioned
	^42^	A 66‐year‐old male	Ustekinumab since 2010	Not mentioned	Symptomatic outpatient with asthenia, anosmia, ageusia	HTN, dyslipidemia and previous Myocardial infarction	Symptom free after 1 month	Maintenance of the remission of psoriasis	No pharmacological treatment	The biologic therapy was interrupted only during the quarantine period, without worsening of psoriasis	Not mentioned
	^42^	A 67‐year‐old female	Adalimumab since 2019	Not mentioned	Asymptomatic outpatient, quarantined for 15 days after contacting family members diagnosed with COVID−19	HTN, metabolic syndrome	Stayed asymptomatic	No change in psoriatic features	Supportive therapy, home rest	Continued biological therapy as formerly prescribed	Not mentioned
	^42^	A 66‐year‐old male	Secukinumab since 2018	Not mentioned	Asymptomatic outpatient, quarantined after continuous contact with his wife mild diagnosed with covid−19	HTN, diabetes mellitus, metabolic syndrome, and obesity (BMI: 32)	Stayed asymptomatic	No change in psoriatic features	Supportive therapy, home rest	Continued biological therapy as formerly prescribed	Not mentioned
	^44^	A 46‐year‐old male	Ustekinumab recently switched to ixekizumab	Moderate–severe chronic plaque psoriasis	Fever, malaise, dyspnea, chest pain, bilateral interstitial pneumonia	Type I Brugada syndrome, arterial HTN	Hospitalized for 22 days, symptom free after 1 month	Not mentioned	Hydroxychloroquine, ceftriaxone noninvasive continuous positive airway pressure (CPAP)	Not mentioned	Not mentioned
	^43^	A 73‐year‐old female	Secukinumab for about 1 year	Chronic plaque psoriasis associated with arthritis (and episodes of dactylitis)	Fever, sore throat, mild dry cough	Hypertension	Symptomatic outpatient	Not mentioned	Hydroxychloroquine	Administered biological therapy during and shortly after the infection	A successful immune response can occur in the presence of IL−17 inhibition
	^45^	A 55‐year‐old female	Apremilast therapy for the last 6 months	Palmoplantar psoriasis	Bilateral pneumonia	Not mentioned	Severe inpatient, Recovered after 1 month	Not mentioned	Not mentioned	Maintained Apremilast during the Hospitalization	
	^45^	A 42‐year‐old male	Apremilast	Psoriasis and psoriatic arthritis	Not mentioned	Not mentioned	Quarantined after continuous contact with his wife with moderate COVID−19 symptoms, after quarantine period, showed no respiratory affections	Not mentioned	Not mentioned	Maintained Apremilast during the confinement	
	^45^	A 55‐year‐old male	Methotrexate, infliximab, switched to apremilast due to recurrent infections and cyclic neutropenia	Plaque and nail psoriasis and psoriatic arthritis	Not mentioned	Hairy cells leukemia	ICU admission with multiple complications such as bacteremia, kidney deterioration, digestive hemorrhages	Not mentioned	Not mentioned	Apremilast was withdrawn in the ICU, topical treatment for psoriasis lesions	
	^45^	A 48‐year‐old female	Secukinumab	Not mentioned	Had the COVID−19 infection criteria (not mentioned in details)	Not mentioned	Outpatient, self‐confined at home	Not mentioned	Not mentioned	Stop receiving secukinumab dose during confinement	
	^45^	A 56‐year‐old male	Infliximab every 6 weeks	Controlled psoriatic arthritis	Bilateral pneumonia, acute respiratory distress	Not mentioned	ICU admission for 15 days	Not mentioned	Not mentioned	Not mentioned	Apremilast can be considered a safe alternative for both infected and uninfected COVID−19 patients
	^45^	A 52‐year‐ old female	Adalimumab therapy failed, then infliximab for the last 3 years	Peripheral spondylarthritis	Moderate inflammatory symptoms	Not mentioned	Symptomatic outpatient that persistency of COVID−19 symptoms forces her to attend the emergency unit on several occasions, until disease recovery	Not mentioned	Not mentioned	Held infliximab	
	^46^	A 73‐year‐old male	Cyclosporine, methotrexate	Uncontrolled psoriasis with severe flare‐ups (manifesting as diffuse erythematous, scaly plaques progressing to erythroderma)	Intermittent fever, malaise, dry cough	Not mentioned	Symptomatic outpatient, Symptoms resolved in 1 week with home rest	Experienced severe psoriasis flare‐ups after the discontinuation of psoriasis treatment during disease course but gradually skin lesions were improved	Hydroxychloroquine, lopinavir/ritonavir combination	Cyclosporine, methotrexate were ceased, Cyclosporine continued 2 weeks after COVID−19 improvement	
	^8^	26 psoriatic patients, mean age of 63.5, 15 male/11 female	Treated with biologics such as Anti TNFa Anti IL−17 Anti‐IL 12/23 Anti IL−23 Mean of duration of therapy=30.7 months (31.6)	Diagnosed with moderate‐to‐severe chronic plaque psoriasis, 8 (31%) of them had joint involvement	Fever (62%), anosmia/Ageusia (27%), cough (19%), dyspnea (8%) Pneumonia (27%), gastrointestinal disorder (11%), other (11%)	Hypertension, other cardiovascular diseases, dyslipidemia, obesity, diabetes, and COPD	42% of patients hospitalized, 73% recovered, 12% sequelae, 23% unknown/pending	Not mentioned	Not mentioned	76% discontinued biologic therapy, and in 27% of patient's biologic therapy were restarted	Findings suggest that the use of biologics is not associated with higher risk of SARS‐COV2 infection or with worse COVID−19 outcome.
	^8^	83‐year‐old male 77‐year‐old male	Under treatment with TNF‐α inhibitors Under treatment with IL‐12/23 inhibitors	Chronic plaque psoriasis	NM	COPD, hypertension, hyperlipidemia, obesity, diabetes Cardiovascular diseases	Both of these patients died because of acute respiratory distress syndrome	NM	NM	NM	
	^51^	77‐year‐old male	Several conventional and biologic drugs, including cyclosporine, methotrexate, infliximab, ustekinumab and secukinumab, and he switched to risankizumab for last 8 months	Chronic plaque psoriasis for 18 years	39°C fever, productive cough with dyspnea, diarrhea, nausea and vomiting	Rheumatoid arthritis, hypertension, acute myocardial infarction 20 years ago, and chronic obstructive pulmonary disease (COPD) for 15 years. A heavy smoker with tobacco consumption	Hospitalized, symptoms ceased after starting the treatment, discharged after 3 weeks in a good clinical condition		Hydroxychloroquine was switched to lopinavir‐ritonavir combination. ceftriaxone and azithromycin for COVID−19‐related pneumonia, but after 6 days, the latter two were replaced by metronidazole due to diarrhea + oxygen support		Treatment with biologic agents, including interleukin−23 (IL−23) inhibitors plays a protective role against COVID−19 infection.
New onset of psoriasis in COVID−19 patients	^49^	A male in his 30 s	Not mentioned	Experiencing pain at the right elbow, together with the appearance of 3 itchy, demarcated erythematous scaly patches on the extensor surface of both elbows and groin	Arthromyalgia, fatigue, diarrhea, anosmia	2 weeks history of painful limitation of the right elbow	Symptomatic outpatient	After 10 days of onset of COVID−19 symptoms skin lesions appeared, then skin and joint symptoms had completely disappeared after 6 weeks	Self‐isolated and self‐medicated with symptomatic drugs	Topical steroids for skin lesions, and nonsteroidal anti‐inflammatory drugs for arthritis	Elevation of interleukin (IL)−17 caused displayed features of reactive psoriatic arthritis
	^50^	62‐year‐old female	History of 3 years of metoprolol, apixaban, beclomethasone, albuterol inhalers, vitamin B12, folate	New onset of palmoplantar Pustules, palmar erythema with hyperkeratosis and desquamation, pink papulopustular lesions on the extremities, and psoriasiform plaques of the trunk and scalp	Fatigue, cough, shortness of breath, night sweats, chills, myalgias	Obesity, asthma, DM, HTN, and atrial fibrillation, 23‐pack‐year history of cigarette smoking (quit 1994) Notable Family history of psoriasis in her aunt and cousin	Not mentioned	Blisters on the palms, pruritic rash continued to spread to involve the forearms, trunk, scalp after 2 weeks resolution of COVID−19symptoms	Not mentioned	Not mentioned	
COVID−19 in known psoriasis patients with specific changes in psoriatic symptoms	^47^	A 38‐year‐old male	Not mentioned	Chronic single active plaque psoriasis affecting the lateral aspect of the right ankle	Fever, dry cough	Not mentioned	Not mentioned	At day 6 following the onset of fever, Flare of guttate psoriasis was occurred as multiple erythematous drop‐like well circumscribed salmon pink erythematous papules with a fine scale, began to form inferior to the knee on the anterior and lateral aspect of the right lower limb	Self‐isolation, recovered after almost a week	Topical readily diluted betamethasone 0.025% cream	
	^48^	A 45‐year‐old male	Methotrexate on and off since 4 years ago, cyclosporine, acitretin	20‐year history of psoriasis with sever erythroderma, ectropion, Severe onycholysis	Fever (Spike pattern 40–40.5°C)	Addicted to amphetamines, opioids, and cigarettes for more than 15 years	Positive COVID−19 PCR along with positive blood culture for staph aureus	Skin, joints movement and general condition improved significantly few days after combination therapy resolution of erythema and scaling in less than 20 days	Cloxacillin, vancomycin, meropenem	Cyclosporine, prednisolone, with reduction of acitretin and then discontinued after 1 week	Remarkable improvement in skin and general condition soon after the administration of cyclosporine and low dose prednisolone
	^52^	A 12‐year‐old male	NM	Diagnosed with plaque psoriasis when he was 5 years old and has been in remission for about 7 years.	High fever, cough	Not mentioned	Not mentioned	New legions; multiple pustular lesions on an erythematous background on the upper extremities and the trunk			COVID−19 might also play a role in the etiopathogenesis of pustular psoriasis through the stimulation of various inflammatory cytokines, especially IL−36.

Abbreviations: BMI, body mass index; DM, diabetes mellitus; HCQ, hydroxychloroquine; HTN, hypertension; ICU, intensive care unit; IL, interleukin; MTX, methotrexate; PCR, polymerase chain reaction; TNF alpha, tumor necrosis factor‐alpha.

## 3 METHOD

We searched Medline database using the following keyboards: “corona virus,” “COVID‐19,” “SARS‐Cov‐2,” “psoriasis,” “erythrodermic psoriasis,” and “psoriasis arthritis” and chose 13 relevant case reports and 6 relevant original articles.

## 4 DISCUSSION

Psoriatic patients mostly rely on immunomodulators. Among immunomodulators, biologics that function through TNF alpha, IL‐23, or IL‐17 inhibition are considered to be the most effective agents in the management of psoriasis.^4^ With the COVID‐19 outbreak, treatments that require immunosuppression or immunomodulation have remained a challenging subject. Although some studies are concerned about the viral replication phase of COVID‐19 infection in patients receiving immunosuppressants, there are other studies that find immunosuppressants beneficial in preventing drastic immune responses when psoriatic patients are afflicted with COVID‐19.^5^, ^6^, ^7^

A lot of studies indicate that the use of biologics does not inflict any further risks compared to the general population and several studies have reported full recovery from COVID‐19 infection in patients receiving biologics. For instance, recently Talamonti et al. published an observational study that included 12,807 patients with moderate‐to‐severe chronic plaque psoriasis treated with biologic agents. Their findings revealed an almost similar incidence rate of mild COVID‐19 among patients in comparison with the general population, which question the association of treatment with biologic agents with a poor outcome or higher susceptibility to COVID‐19 infection in psoriatic patients.^8^ Consistent with Talamonti findings, certain studies like Brownstone et al. and Messina et. al. not only have reported successful recovery in biologic receiving patients but also suggest that biologics could be considered as potential life savers against COVID‐19 induced cytokine storm.^9^, ^10^

Another type of immunosuppressants that are regularly prescribed for psoriasis is nonbiologics, which include methotrexate (MTX) and cyclosporine.^11^, ^12^ Existing studies advice against the use of these agents for it, which has been demonstrated that MTX is accompanied with up to 45% increased risk of COVID‐19 infection. On the contrary, some studies find the anti‐inflammatory effects of MTX desirable in the inflammatory phase of COVID‐19 infection.^13^, ^14^

A case report has looked into 4 cases that were in close contact and 3 out of 4 of them were infected with COVID‐19 but the fourth one that was under treatment with MTX because of psoriasis did not become infected.^15^ Although cyclosporine has been reported to be safer than MTX in the course of COVID‐19 infection but still a number of studies suggest abstain from it.^16^

Another challenging aspect of this matter is that COVID‐19 infection itself provokes immune responses that can be progressed to cytokine storm resulting in a hyperinflammation state in the body that attributes to the pathogenesis of psoriasis, which similarly causes hyperinflammation.^17^ This immense cytokine release might exacerbate psoriatic patients' condition as it has been reported by different studies.^18^, ^19^ We have discussed in our previous studies about the potential association between COVID‐19 and dermatology conditions.^6^, ^19^, ^20^, ^21^, ^22^, ^23^, ^24^, ^25^, ^26^, ^27^, ^28^, ^29^, ^30^, ^31^, ^32^, ^33^

Using glucocorticoids is one of the main therapeutic strategies in patients with severe COVID‐19 symptoms; in psoriatic patients, the administration of glucocorticoids during the course of COVID‐19 infection might affect the patient's psoriasis presentation, as in some cases early after the administration of steroids, psoriatic lesions seem to be mitigated, and after finishing the course of treatment, some patients have reported to experience certain degrees of psoriasis flare‐up.^6^, ^19^, ^20^, ^21^, ^22^, ^25^, ^26^, ^27^, ^28^, ^29^, ^30^, ^31^

Currently, there is no general consensus about the medications used in COVID‐19 treatment but from the beginning of the pandemic Hydroxychloroquine (HCQ) has been one of the most common choices in COVID‐19 treatment.^34^ Certain case reports have suggested HCQ to have worsening effects on the course of psoriasis. Although the exact cause of this effect is not entirely clear, some studies attribute this to an elevation of IL‐17 in patients taking HCQ.^35^

Sachdeva et al. have reported 9 cases with a new onset of psoriasis after taking HCQ.^36^ Although they hold HCQ responsible for this, a handful of case reports exist on the topic of new psoriasis onsets after COVID‐19 infection, which conclude these new onsets to be the result of the infection itself.^35^ Lehmann et al. have even reported a case with a new onset of psoriasis after COVID‐19 vaccination.^37^

Another commonly used medication in COVID‐19 is azithromycin. Although the benefits of this macrolide in COVID‐19 treatment have remained controversial, it is considered to have a potential immunomodulatory effect on keratocytes and epidermal Langer Hans cells, which could be helpful in the improvement of psoriatic legions.^38^ But data on this matter are very limited and further investigation is crucial.

As it was mentioned, our first case had consumed amoxicillin as well. Although there is existing evidence about the association between consumption of some antibiotics like tetracycline and psoriasis aggravation, flare‐ups following derivatives of penicillin administration have been reported to be extremely rare. Therefore, we assume that amoxicillin may not have been very likely to be the culprit that caused the exacerbation.^39^

Both of our cases had stopped their immune suppressants before being infected with COVID‐19, and they both had consumed HCQ. Although the main cause of their flare‐up is not entirely clear but according to previous studies, we can assume that stopping their immunosuppressive therapies without consulting their doctors might have made them more susceptible to the COVID‐19‐related cytokine storm; moreover, the use of HCQ might have aggravated their underlying psoriasis.

With the combination of limited access to their medications due to the COVID‐19 outbreak, inflammation caused by COVID‐19 infection and the adverse effects of the medications currently in use for opposing COVID‐19 infection, adding to it the stress that the COVID‐19 causes (which is considered another factor in psoriasis aggravation), chronic disease patients, and their doctors are faced with a dilemma for finding a sweet spot of managing their patients' underlying psoriasis condition and overcoming the infection caused by COVID‐19.^40^

Overall, evidence suggests that discontinuing the immunosuppressive therapies in patients who had been using them before being infected with COVID‐19 is not advisable, while more evidence points to biologics being safe but even nonbiologics that are controversial for their safety are recommended to be continued with caution.

To have a better understanding of the clinical aspects and managements of psoriasis in the COVID‐19 pandemic, we have reviewed and summarized reported cases of psoriasis patients diagnosed with COVID‐19 and cases with new onset of psoriasis in COVID‐19 cases. We categorized case reports into three main groups.

### 4.1 COVID‐19 infection in known psoriasis patients without specific changes in psoriatic symptoms

A number of studies have reported an asymptomatic course of COVID‐19 infection in psoriatic patients that were on biologic treatment without any changes in their psoriasis condition. Balestri et al. and Conti et al. have reported patients that were unaware of their COVID‐19 infection until their close family members were tested positive and they remained symptom free in the whole duration of their COVID‐19 infection.^41^, ^42^ Benhadou et al reported a 40‐year‐old woman with mild COVID‐19 symptoms that were worsening whereat she received her scheduled Guselkumab and her respiratory symptoms were instantly mitigated.^5^ There were also other cases including the cases reported by Brownstone et al. and Galluzzo et al. and 2 of the cases reported by Conti et al. that developed mild COVID‐19 symptoms and recovered without being hospitalized.^9^, ^42^, ^43^ In some cases, the course of COVID‐19 was more challenging; for example, in one of the cases reported by Conti et al. the patient was admitted to intensive care unit (ICU), but fortunately he also successfully recovered from the infection. ICU admission was reported by Queiro et al. as well. Facheris et al. also reported a 22‐day hospitalization in a 46‐year‐old male.^44^, ^45^ The aforesaid cases were all receiving biologics, and no alteration in the course of their psoriasis was reported. Nasiri et al. reported a patient under treatment with MTX and cyclosporine with uncontrolled psoriasis and intermittent severe flare‐ups that also recovered without being hospitalized.^46^ However, his psoriatic legions were worsened at first because he had discontinued his immunosuppressive medications, but then his legions gradually improved.

### 4.2 COVID‐19 infection in known psoriasis patients with specific changes in psoriatic symptoms

Gananandan et al. reported a 38‐year‐old male with chronic active psoriatic plaques that 6 days after the onset of fever caused by COVID‐19 developed multiple erythematous legions on his lower limbs.^47^

In Ghalamkarpour's paper, a 45‐year‐old male with a 20‐year history of psoriasis had erythrodermic flare‐up following COVID‐19 infection. Moreover, the patient had discontinued his psoriasis medications before being infected with COVID‐19.^48^

### 4.3 New onset of psoriasis in COVID‐19 patients

De Stefano et al. reported a male in his thirties without any previous history of psoriasis that was infected with COVID‐19 with mild symptoms, but 10 days after recovery from COVID‐19, psoriatic legions started appearing from his groin and the extensor surface of his elbows.^49^

Another similar case was a 62‐year‐old female reported by Mathieu et al. that developed psoriatic legions 2 weeks after COVID‐19 recovery.^50^ Another remarkable case was reported Lehmann et al. describing a new onset of guttate psoriasis after COVID vaccination.^37^

**SUMMARY**

- Continuation of immunosuppressive treatments in patients that become infected with COVID‐19 is a matter of controversy.
- COVID‐19 causes cytokine storm in the body that might aggravate diseases with an inflammation‐based pathophysiology.
- Current information is still limited and controversial about the dynamic association between COVID‐19 and pre‐existing immune‐mediated diseases (including psoriasis) in terms of the impact of COVID‐19 infection and its treatments on the course of the underlying disease and the effect of the pre‐existing disease and its treatments on the COVID‐19 infection. Although a number of studies and recommendations about this concern exist but since there is still no general agreement, further investigation is crucial.
- When a patient with a pre‐existing immune‐mediated disease is infected with COVID‐19, cessation or alteration of the routine medications, the cytokine storm caused by the infection or the effects of COVID‐19 medications might change the course of the underlying disease and flare‐ups might occur. What this study adds?
- Patients who had discontinued their psoriasis medications prior or after being afflicted with COVID‐19 were more likely to experience severe psoriasis flare‐ups.
- Patients who had not missed their scheduled biologic administration were likely to experience mild COVID‐19 symptoms while their psoriasis condition remained unchanged.
- Biologics might potentially help with COVID‐19 infection recovery.
- Morbidity and mortality in psoriatic patients were not higher than the general population.
- Evidence suggests that biologics are safe in the course of COVID‐19 infection and patients who had stopped their scheduled injection were more likely to experience flare‐up, therefore, withholding biologics due to COVID‐19 infection is not advisable.
- Although the use of non‐biologics in the pandemic era is more controversial but some studies suggest that like biologics, non‐biologics can also have desirable effects on overcoming the hyperinflammation state caused by COVID‐19.
- There are substantial controversies about starting non‐biologics in patients at the time of being infected with COVID‐19 but a lot of studies suggest continuing non‐biologics in patients who were receiving them before being infected with COVID‐19.
- Using Glucocorticoids is one of the main therapeutic strategies in patients with severe COVID‐19 symptoms; in psoriatic patients, administration of glucocorticoids during the course of COVID‐19 infection might affect the patient’s psoriasis presentation, as in some cases early after the administration of steroids, psoriatic lesions seem to be mitigated and after finishing the course of treatment, some patients have reported to experience certain degrees of psoriasis flare‐up.

## 5 CONCLUSION

Although the main reason for their flare‐up in not entirely clear in the two cases that we reported but we assume that discontinuation of psoriasis systemic treatment and not receiving any immunosuppressant had made them more susceptible to the COVID‐19‐related cytokine storm and disease flare‐up. Our literature review revealed that COVID‐19 in psoriatic patients is not accompanied with any further morbidity and mortality compared to the general population. Patients that had discontinued their psoriasis medications were more likely to experience psoriasis flare‐ups, but patients that maintained their routine medications were less likely to experience any alterations in their underlying psoriasis. A considerable number of studies suggest that biologics seemed to have helped psoriatic patients mitigate their COVID‐19 symptoms and accelerated their recovery from COVID‐19. Some studies suggest that taking HCQ might aggravate the underlying psoriasis condition.

Most flare‐up cases were in patients that were not receiving biologics and had discontinued their nonbiologic immunosuppressive medications, whether this has been due to the COVID‐19‐related cytokine storm or the medications given to them for COVID‐19 is not clear but when the underlying psoriasis is neglected, COVID‐19 is more likely to trigger flare‐up.

## CONFLICT OF INTEREST

The authors certify that there is no conflict of interest with any financial organization regarding the material discussed in the manuscript.

## AUTHOR CONTRIBUTION

EB, ASB, and AG performed the research; EB and ASB designed the study. MHFK contributed essential reagents or tools; NS, ZS, MHFK, and AG searched the literature. NS, ZS, and ASB wrote the paper; all authors contributed in revising the paper critically for important intellectual content; EB, ASB, and AG wrote the paper; and all authors have read and approved the final manuscript.

## CONSENT

Written informed consent was obtained from the patients to publish this report.

## Data Availability

Data are available on reasonable request from the corresponding author.
